# Meta-Transcriptomic Comparison of the RNA Viromes of the Mosquito Vectors *Culex pipiens* and *Culex torrentium* in Northern Europe

**DOI:** 10.3390/v11111033

**Published:** 2019-11-06

**Authors:** John H.-O. Pettersson, Mang Shi, John-Sebastian Eden, Edward C. Holmes, Jenny C. Hesson

**Affiliations:** 1 Department of Medical Biochemistry and Microbiology/Zoonosis Science Center, Uppsala University, 75237 Uppsala, Sweden; Jenny.Hesson@imbim.uu.se; 2 Marie Bashir Institute for Infectious Diseases and Biosecurity, Charles Perkins Centre, School of Life and Environmental Sciences and Sydney Medical School, the University of Sydney, Sydney, New South Wales, NSW 2006, Australia; mang.shi@sydney.edu.au (M.S.); edward.holmes@sydney.edu.au (E.C.H.); 3 Centre for Virus Research, The Westmead Institute for Medical Research, Sydney, NSW 2145, Australia

**Keywords:** mosquito-borne RNA viruses, evolution, ecology, meta-transcriptomics, RNA-sequencing, *Culex* mosquitoes

## Abstract

Mosquitoes harbor an extensive diversity of ‘insect-specific’ RNA viruses in addition to those important to human and animal health. However, because most studies of the mosquito virome have been conducted at lower latitudes, little is known about the diversity and evolutionary history of RNA viruses sampled from mosquitoes in northerly regions. Here, we compared the RNA virome of two common northern mosquito species, *Culex pipiens* and *Culex torrentium*, collected in south-central Sweden. Following bulk RNA-sequencing (meta-transcriptomics) of 12 libraries, comprising 120 specimens of *Cx. pipiens* and 150 specimens of *Cx. torrentium*, we identified 40 viruses (representing 14 virus families) of which 28 were novel based on phylogenetic analysis of the RNA-dependent RNA polymerase (RdRp) protein. Hence, we documented similar levels of virome diversity as in mosquitoes sampled from the more biodiverse lower latitudes. Many viruses were also related to those sampled on other continents, indicative of a widespread global movement and/or long host–virus co-evolution. Although the two mosquito species investigated have overlapping geographical distributions and share many viruses, several viruses were only found at a specific location at this scale of sampling, such that local habitat and geography may play an important role in shaping viral diversity in *Culex* mosquitoes.

## 1. Introduction

The mosquito genus *Culex* (Diptera; *Culicidae*) comprises more than a thousand species, with representatives found globally [1]. *Culex* species are vectors of a number of important pathogens including West Nile virus (WNV) (*Flaviviridae*), Japanese encephalitis virus (JEV) (*Flaviviridae*), and Sindbis virus (SINV) (*Togaviridae*), as well as a variety of nematodes [1,2,3]. One of the most widespread *Culex* species is the Northern House mosquito, *Cx. pipiens*, which is distributed across the northern hemisphere. In Europe and the Middle East, it occurs together with *Cx. torrentium*, another *Culex* species with females and larvae that are morphologically identical to *Cx. pipiens*. These two species have overlapping distributions and share larval habitats. However, *Cx. torrentium* dominates in Northern Europe while *Cx. pipiens* is more abundant in the south [4]. Both species are vectors for a number of bird-associated viruses that can cause disease in Europe, such as WNV, that may cause a febrile disease with encephalitis, and SINV that may result in long-lasting arthritis [2,5]. *Cx. pipiens* is one of the most common WNV vectors in both Southern Europe and North America, while *Cx. torrentium* is the main vector of SINV in Northern Europe [2,6]. Infections with these pathogenic viruses occur in late summer when viral prevalence increases in passerine birds, the vertebrate hosts of both of these viruses [7,8]. Despite their importance as vectors, little is known about the detailed biology of *Cx. pipiens* and *Cx. torrentium* due to the difficulties in species identification, which can only be reliably achieved through molecular means. Much of the biology of these species, such as their larval habitat and feeding preferences, is considered similar. However, one important difference between the two species is that while *Cx. pipiens* harbors a high prevalence of the intracellular bacteria *Wolbachia pipientis*, it is seemingly absent in *Cx. torrentium* [9].

In recent years, studies utilizing RNA-sequencing (RNA-Seq, or ‘meta-transcriptomics’) have revealed an enormous RNA virus diversity in both vertebrates and invertebrates [10,11]. Mosquitoes are of particular interest as many are well-known vectors of pathogenic viruses. Importantly, these pathogenic viruses represent only a fraction of the total virome in the mosquito species investigated. Indeed, mosquitoes clearly carry a large number of newly described and divergent arthropod-specific viruses, with representatives from many genetically diverse virus families and orders, such as the *Flaviviridae*, *Togaviridae*, and the *Bunyavirales* [12,13,14,15,16]. However, most studies have been conducted on latitudes below 55°, such that there is a marked lack of data of the mosquito viral diversity present in northern temperate regions where the composition of mosquito species as well as environmental parameters differ significantly from lower latitudes, and where human populations are at high density. In addition, for many life forms, biodiversity increases towards the equator [17], and the species richness of mosquitoes is greater in tropical regions than temperate regions [18]. A central aim of the current study was therefore to investigate whether viral diversity co-varies in the same manner. Given that *Cx. pipiens* and *Cx. torrentium* are two common *Culex* species in Northern and Central Europe, and known vectors of SINV and WNV, they were chosen for RNA virome investigation and comparison by RNA-Seq.

## 2. Materials and Methods 

### 2.1. Mosquito Collection 

Mosquitoes were collected from two regions in Sweden: (i) from floodplains of the Dalälven River in central Sweden (60.2888; 16.8938) in 2006, 2009, and 2011; and (ii) around the city of Kristianstad, in southern Sweden (56.0387; 14.1438) in 2006 and 2007. Collections were performed using Centers for Disease Control and Prevention-light traps baited with carbon dioxide, and catches were sorted and identified to species on a chilled table, using keys by Becker et al. [19]. In total, legs from 270 *Cx. pipiens/torrentium* mosquitoes were removed to enable molecular identification to species [20]. Bodies were homogenized in phosphate-buffered saline buffer supplemented with 20% fetal calf serum and antibiotics and stored at –80 °C until further processing.

### 2.2. Sample Processing and Sequencing

Total RNA was extracted from 12 pools from the homogenate of individual *Cx. torrentium* (*n* = 150) and *Cx. pipiens* mosquitoes (*n* = 120) (Table S1), using the RNeasy^®^ Plus Universal kit (Qiagen, Hilden, Germany) following the manufacturer’s instructions. Three pools, L1 and L2 for *Cx. torrentium* and L3 for *Cx. pipiens*, respectively, were previously shown to be positive for SINV and were included as a reference for RNA viral diversity in the presence of a pathogen. The extracted RNA was subsequently DNased and purified using the NucleoSpin RNA Clean-up XS kit (Macherey-Nagel, Düren, Germany). Prior to library construction, ribosomal RNA (rRNA) was depleted from the purified total RNA using the Ribo-Zero Gold (human–mouse–rat) kit (Illumina, San Diego, CA, USA) following the manufacturer’s instructions. Sequencing libraries were then constructed for all rRNA-depleted RNA-samples using the TruSeq total RNA library reparation protocol (Illumina). All libraries were sequenced on a single lane (paired-end, 150 bp read-length) on an Illumina HiSeq X10 platform. Library preparation and sequencing was carried out by the Beijing Genomics Institute, Hong Kong. All 12 libraries were quality trimmed with Trimmomatic v.0.36 [21], using default settings for paired-end sequence data, and then assembled de novo using Trinity v.2.5.4 [22], employing the default settings with read normalization.

### 2.3. Identification of Viruses and Wolbachia Bacteria

Trinity assemblies were screened against the complete non-redundant NCBI GenBank nucleotide (nt) and protein (nr) databases using blastn, primarily to identify closely related RNA viruses and false-positive host-derived hits, as well as a diamond [23] blastx analysis primarily to identify divergent RNA viruses, with cut-off e-values of 1 × 10^−5^ in both cases. To determine whether some of the assemblies represent potential endogenous viral elements (EVEs), all virus viral assemblies were blasted against the *Culex quinquefasciatus* reference genome (GCA_000209185.1). Assemblies identified as RNA viruses were screened against the NCBI Conserved Doman Database with an expected value threshold of 1 × 10^−3^ to identify viral sequence motifs. The mitochondrial cytochrome c oxidase I (COX1) gene, mined from the sequence data, and all contigs with RdRp-motifs was mapped back to all quality trimmed libraries to estimate abundance using Bowtie2 [24], employing the default local setting. A virus was considered to be in high abundance if: (i) it represented >0.1% of total ribosomal-depleted RNA reads in the library, and (ii) if the abundance was higher to that of the abundant host COX1 gene [12,25]. Such high abundance viruses were tentatively assumed to be mosquito associated. Hits below the level of cross-library contamination due to index-hopping, measured as 0.1% of the most abundant library for the respective virus species or less than 1 read per million mapped to a specific virus contig, was considered negative (colored grey in Table 1 and Table 2, respectively). To investigate the presence of *Wolbachia* bacteria in the libraries, published sequences of the *Wolbachia Cx. pipiens* wsp surface protein gene (DQ900650.1) and the mitochondrial COX1 gene (AM999887.1) were mapped backed against all libraries using the above criteria for abundance and presence/absence.

### 2.4. Inference of Virus Evolutionary History and Host Associations 

The evolutionary (i.e., phylogenetic) history of the viruses discovered were inferred by aligning protein translated open reading frames with representative sequences from the *Alphaviridae*, (Order) *Bunyavirales*, *Endornaviridae*, *Luteoviridae*, (Order) *Mononegavirales*, Nido-like viruses, (Order) *Orthomyxovirales*, *Partitiviridae*, *Picornaviridae*, Qin-like viruses, *Reoviridae*, *Totiviridae*, *Tymoviridae* and *Virgaviridae* and Negev-like viruses. All RdRp amino acid sequence alignments were performed using the E-INS-i algorithm in Mafft [26]. Poorly aligned regions, in which amino acid positional homology could not be confirmed, were then removed from the alignments using TrimAl utilizing the ‘strict’ settings. Finally, phylogenetic trees were computed with a maximum likelihood approach as implemented in PhyML [27] employing the LG+Γ model of amino acid substitution, Sub-tree Pruning and Re-grafting branch-swapping and the approximate likelihood ratio test (aLRT) with the Shimodaira–Hasegawa-like procedure used to assess branch support. The resultant phylogenetic trees were edited and visualized with FigTree v.1.4.2. 

To help assess whether the novel viruses discovered were likely mosquito associated, that is, to distinguish those that actively replicate in the host from those present in diet mosquito or a co-infecting micro-organism, we considered four factors: (i) the abundance of viral contigs per total number of reads in a library (i.e., >0.01% was considered abundant); (ii) if the abundance of the virus was higher in relation to the host COX1 gene; (iii) presence in the individual sequencing libraries (i.e., present = yes, not present = no); and (iv) clear phylogenetic clustering with other mosquito-derived viruses (i.e., clustered/did not cluster with other mosquito associated viruses = yes/no). A mosquito association was tentatively assigned if a particular virus met two or more of the four criteria.

The raw sequence data generated here have been deposited in the NCBI Sequence Read Archive (BioProject: PRJNA516782) and all viral contigs have been deposited on NCBI GenBank (accession numbers: MK440619–MK440659).

## 3. Results

### 3.1. RNA Virome Characterization

We characterized the RNA viral transcriptome of two mosquito species, *Cx. pipiens* and *Cx. torrentium,* collected from central and southern Sweden (Table S1). After high-throughput sequencing, a total of 569,518,520 (range 34,150,856–62,936,342) 150bp reads were produced from 12 ribosomal RNA-depleted sequence libraries that were assembled into 153,583 (4333–33,893) contigs. From all the contigs assembled, we identified 40 that contained RdRp sequence motifs and hence indicative of viruses, belonging to 14 different viral families/orders: *Alphaviridae*, *Bunyavirales*, *Endornaviridae*, *Luteoviridae*, *Mononegavirales*, *Nidovirales*, *Orthomyxoviridae*, *Partitiviridae*, *Picornaviridae*, *Reoviridae*, *Totiviridae*, *Tymoviridae*, and representatives from the divergent *Virgaviridae*, *Negeviridae*, and Qin viruses. Following a similarity search of all virus sequences against a *Cx. quinquefasciatus* reference genome we found no evidence that any of the discovered viruses were derived from the mosquito host genome (i.e., present as EVEs). For each viral family/order, between one and five virus species were identified and in total 28 novel RNA virus species were discovered here, which were named based on geographical location.

**Figure 1:**
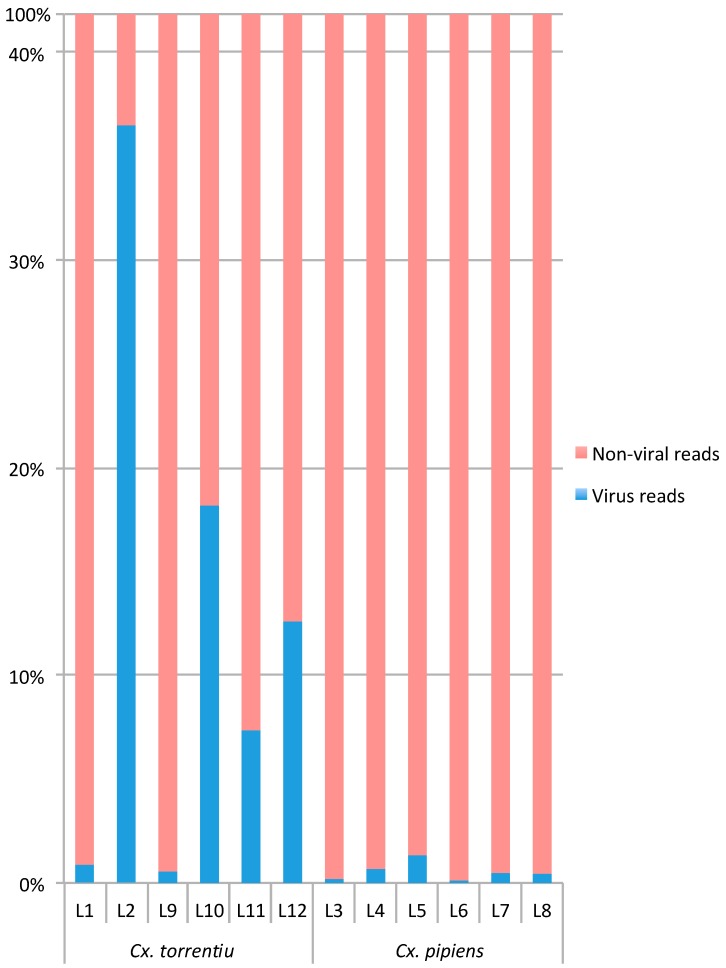
Estimation of virome composition in comparison to host (non-viral) content in each library.

The relative number of all virus reads, as mapped to contigs with RdRp-motifs, compared to the total amount of non-viral ribosomal RNA-depleted reads per library varied between 0.1%–36.6% (Table 1). Notably, libraries 2, 10, 11, and 12 from *Cx. torrentium* were characterized by a higher number of viral reads compared to non-viral reads (Figure 1, Table 1). The individual abundance of each viral species, measured as the number of reads mapped to each RdRp contig divided by the total amount of ribosomal RNA-depleted reads in the library ×1000,000 (i.e., reads per million, RPM), varied between 1.09–10,006.67 RPM for *Cx. pipiens* and 1.08–303,145.83 RPM for *Cx. torrentium*. In comparison, the abundance of host reads, as measured by the presence of the host mitochondrial protein COX1, was more stable and varied only between 4.22–66.99 RPM across all libraries (Table 2).

### 3.2. Virome Comparison between Mosquito Species and Geographical Regions

**Figure 2:**
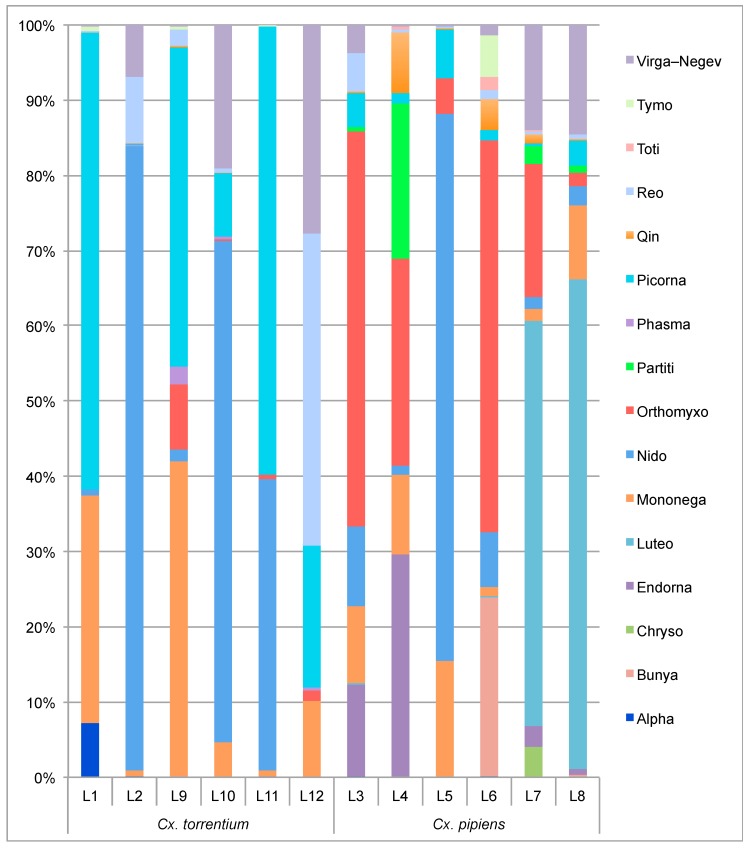
Comparison of the virome family composition and abundance between *Cx. pipiens* and *Cx. torrentium*. For ease of presentation, abbreviations are used to indicate virus taxonomy in each case. The viral families represented in each bar are shown according to the family order in the legend. Abbreviations: Alpha = *Alphaviridae*; Bunya = *Bunyaviridae*; Chryso = *Chrysoviridae*; Endorna = *Endornaviridae*; Luteo = *Luteoviridae*; Mononega = Mononegavirales; Nido = Nido-like viruses; Orthomyxo = Orthomyxovirales; Partiti = *Partitiviridae*; Phasma = Phasmavirus (*Bunyaviridae*); Picorna = *Picornaviridae*; Qin = Qin-like viruses; Reo = *Reoviridae*; Toti = *Totiviridae*; Tymo = *Tymoviridae*; Virga–Negev = *Virgaviridae* and Negev-like viruses.

**Figure 3:**
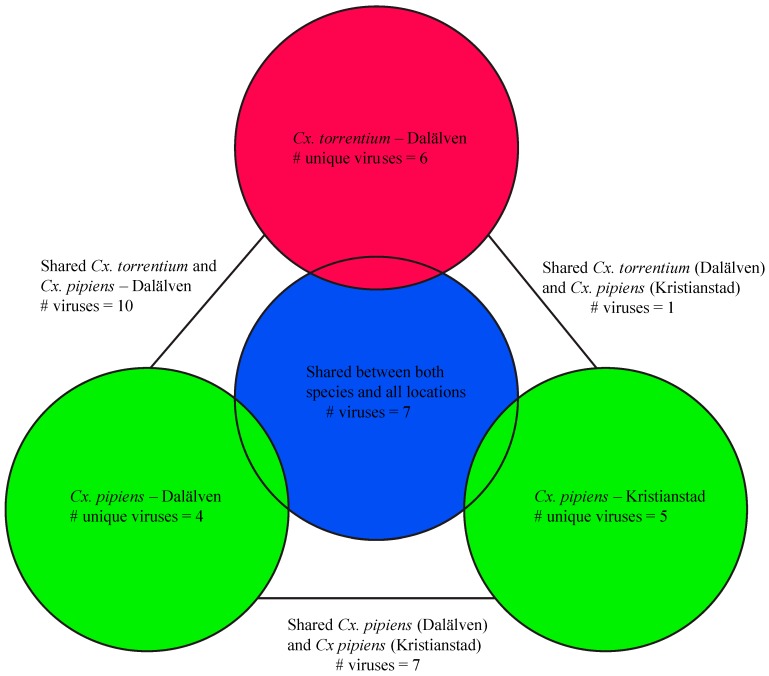
Venn diagram showing the number of unique and shared viruses per location per mosquito species.

Both the composition and abundance of the virus species and families observed seemingly differed between the two mosquito species (Figure 2, Table 2). Of the 40 newly discovered virus species, most were found in *Cx. pipiens* which harbored 34 species: 23 of these are newly described in *Cx. pipiens* and 11 have been described previously. Sixteen of these 34 virus species were unique to *Cx. pipiens* and hence not present in *Cx. torrentium*. Similarly, 24 of the 40 virus species were discovered in *Cx. torrentium*: 18 of these were newly described in *Cx. torrentium* and six have been described previously. Six viruses found in *Cx. torrentium* were not present in *Cx. pipiens* (Figure 3, Table 2). 

**Figure 4:**
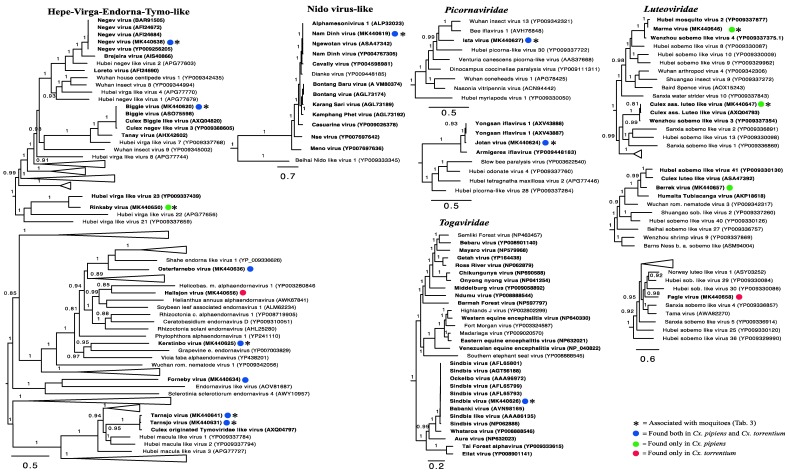
Phylogenetic analysis of all the positive-sense RNA viruses identified here (marked by colored circles) along with representative publicly available viruses. Those viruses most likely associated with mosquitoes are marked by an *. Numbers on branches indicate Shimodaira–Hasegawa (SH) support, and only branches with SH support ≥80% are indicated. Branch lengths are scaled according to the number of amino acid substitutions per site. All phylogenetic trees were midpoint-rooted for clarity only. Known mosquito-associated viruses and virus sequences that were derived from mosquito samples are indicated in bold.

**Figure 5:**
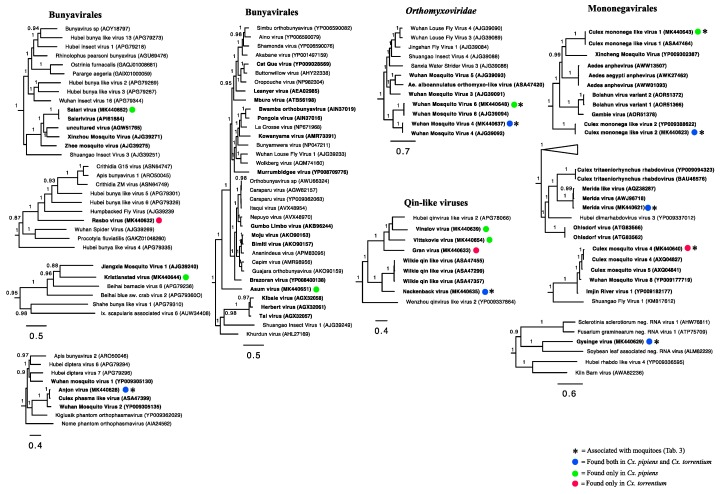
Phylogenetic analysis of all the negative-sense RNA viruses identified here (marked by colored circles) along with representative publicly available viruses. Those viruses most likely associated with mosquitoes are marked by an *. Numbers on branches indicate SH support, and only branches with SH support ≥80% are indicated. Branch lengths are scaled according to the number of amino acid substitutions per site. All phylogenetic trees were midpoint-rooted for clarity only. Known mosquito-associated viruses and virus sequences that were derived from mosquito samples are indicated in bold.

**Figure 6:**
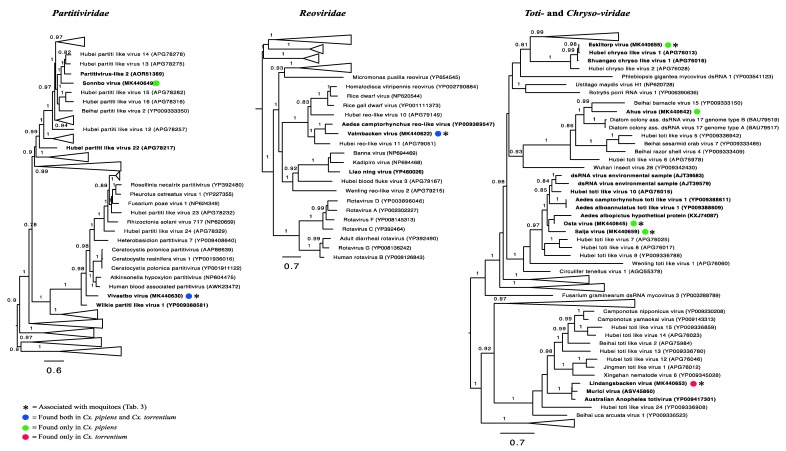
Phylogenetic analysis of all the double-stranded RNA viruses identified here (marked by colored circles) along with representative publicly available viruses. Those viruses most likely associated with mosquitoes are marked by an *. Numbers on branches indicate SH support, and only branches with SH support ≥80% are indicated. Branch lengths are scaled according to the number of amino acid substitutions per site. All phylogenetic trees were midpoint-rooted for clarity only. Known mosquito-associated viruses and virus sequences that were derived from mosquito samples are indicated in bold.

We next analyzed potential host relationships by comparing the abundance (total abundance, of which >0.01% was considered abundant, and in relation to the host COX1 gene), presence across multiple libraries, and phylogenetic relationship to other viruses (Table 3). If a particular virus met two or more of the four criteria, it was tentatively considered as mosquito associated. These data suggest that 16 of the 40 viruses were likely mosquito associated, of which one and two were unique to *Cx. pipiens* and *Cx. torrentium*, respectively (Figure 4, Figure 5 and Figure 6). The host association was unclear in the remaining viruses (for example, they could be associated with micro-organisms co-infecting the mosquitoes) and hence could not be safely assumed to infect mosquitoes. For example, Ahus virus (*Totiviridae*) was at low abundance, was not present in several libraries, and clustered with viruses derived from various environmental samples, suggesting that it is not mosquito associated. Similarly, although Gysinge virus (*Mononegavirales*) was abundant and present in several libraries (Table 3), its closest relative (Figure 5) was a soybean leaf-associated virus [28] which means that its proposed mosquito association is uncertain and clearly needs to be investigated further. Conversely, Culex mononega-like virus 2 (Figure 5) was abundant, present in several libraries and clustered with other mosquito viruses, suggesting that it is mosquito associated. All potential mosquito host association data is summarized in Table 3.

Notably, *Cx. torrentium* carried four viruses of markedly higher abundance compared to *Cx. pipiens*: (i) Nam Dinh virus (303,145 RPM, or 42% of all viral reads and more than 30% of all (non rRNA) reads, respectively, in library L2); (ii) Biggie virus (35,063 RPM, or 4.6% of all viral reads and 3.5% of all reads, respectively, in library L12); as well as two newly identified viruses, (iii) Valmbacken virus (52,264 RPM, or 27% of all viral reads and 3.5% of all reads, respectively, in library L12); and (iv) Jotan virus (41,738 RPM, or 56% of all viral reads and 4.2% of all reads, respectively, in library L11) (Table 2, Table S2). *Cx. pipiens* was characterized by a slightly higher abundance of orthomyxo-like and luteoviruses compared to *Cx. torrentium* (Figure 2), although in both mosquito species the most abundant virus was the Nam Dinh virus that reached 10,006 RPM (or 73% of all viral reads and 1% of all reads, respectively) in library L5.

We next compared the virome composition in the sampled mosquitoes between Kristianstad in the south and the floodplains of the Dalälven River situated roughly 600 km further north. In the case of *Cx. pipiens* this analysis revealed a total of 20 virus species from Kristianstad, 12 of which were unique to *Cx. pipiens* and five detected in Kristianstad only, all of which were unique to *Cx. pipiens*: Asum virus (*Bunyaviridae*), Eskilstorp virus (Chrysoviridae), Kristianstad virus (*Bunyaviridae*), Rinkaby virus (Virga–Negev virus), and Vittskovle virus (*Qinvirus*). A total of 28 viruses were found in *Cx. pipiens* from Dalälven. Eleven of these were unique to *Cx. pipiens* and four were unique to *Cx. pipiens* from Dalälven: Salari virus (*Bunyavirales*), Sonnbo virus (*Partitiviridae*), Culex mononega-like virus 1 (*Mononegavirales*), and Berrek virus (*Luteoviridae*) (Table 2, Figure 2, Figure 4, Figure 5 and Figure 6). A similar relationship was found for *Cx. torrentium*. In the case of Dalälven, 24 viruses were found in *Cx. torrentium*, of which 18 were shared with *Cx. pipiens* and six of which were unique to *Cx. torrentium* (Table 2, Figure 2, Figure 4, Figure 5 and Figure 6). Hence, the majority of the mosquito viruses identified here were shared both between species and geographical regions, even though only 30 specimens of *Cx. pipiens* were available from Kristianstad. In contrast, we found several virus species that were unique to a specific location, which could be indicative of virome differentiation at a local geographic scale, although this will need to be confirmed with additional sampling.

### 3.3. Evolutionary History and Host Associations of the Discovered RNA Viruses

Our phylogenetic analyses of the viruses newly identified here showed that several were closely related to previously identified viruses, and that many form clusters with mosquito associated and/or *Culex* associated viruses within particular viral families, such as the Merida virus and Gysinge virus (*Mononegavirales*), and Tarnsjo virus (*Tymovirales*) (Figure 4, Figure 5 and Figure 6). In contrast, other novel viruses clustered with those neither associated with mosquitoes nor other arthropods: that they are distinguished by long branches suggests that they might infect diverse host taxa. 

### 3.4. Positive-Sense RNA Viruses

We identified 16 positive-sense RNA viruses, of which 12 were likely novel. The majority fell within the Hepe–Virga–Endorna–Tymo-like virus complex (*n* = 8), whereas the others fell within *Nidovirales* (*n* = 1), *Luteoviridae* (*n* = 4), *Picornavirales* (*n* = 2), and *Togaviridae* (*n* = 1), respectively (Figure 4). The viruses discovered contain those that are closely related to other mosquito-associated viruses, such as the highly abundant Nam Dinh virus (*Nidovirales*), as well as those without clear host associations. For example, Biggie virus clusters in a distinct group of Biggie viruses (*Virga/Endorna-viridae*) sampled from other *Culex* mosquitoes [15]. We also identified several novel and divergent viruses in the *Endornaviridae*—specifically the Kerstinbo virus and Hallsjon virus—that do not cluster with other arthropod-associated viruses (Figure 4). Similarly, within the *Tymoviridae* we detected two variants of a *Culex*-associated virus, Tarnsjo virus, that are closely related to a *Culex* associated Tymoviridae-like virus [15].

We identified four viruses within the *Luteoviridae*: *Culex* associated luteo-like virus, as well as the novel Berrek, Fagle, and Marma viruses. *Culex* associated luteo-like virus was previously found in a pool of *Culex* sp. mosquitoes from North America [15]. Both the newly discovered Berrek virus and Marma virus grouped with other luteoviruses found in mosquitoes (Figure 4), but only the Marma virus was abundant, suggesting that it is *Culex* associated (Table 2, Table 3).

Two novel picornaviruses were also identified. The abundant Rinkaby virus clustered with Yongsan iflavirus 1 virus, sampled from *Cx. pipiens* mosquitoes from South Korea and was therefore considered a bona fide *Culex* associated picornavirus. Although the Ista virus did not cluster with viruses derived from mosquitoes, its high abundance and presence in all libraries (Figure 4, Table 3) suggest that it is also *Culex* associated. 

Finally, four of our libraries—L1 and L2 for *Cx. torrentium* and L3 and L6 for *Cx. Pipiens*—contained reads for SINV. Importantly, whereas the presence of SINV could be confirmed with PCR in library L1, L2 and L3, it was not PCR confirmed in L6 such that contamination cannot be excluded in this case. 

### 3.5. Negative-Sense RNA Viruses

In total, we identified 16 negative-sense RNA viruses, including nine novel viruses: *Bunyavirales* (*n* = 5), *Mononegavirales* (*n* = 5), Qin-like viruses (*n* = 4), and *Orthomyxoviridae* (*n* = 2) (Figure 5). As was the case for the positive-sense RNA viruses, some of these viruses have been identified previously and clustered with viruses found in mosquitoes of the same genera, including Salari virus (*Bunyavirales*) and a number of novel viruses such as Anjon virus (*Bunyavirales*).

Within the order *Mononegavirales*, Merida virus, *Culex* mononega-like virus 2, *Culex* mosquito virus 4, and *Culex* mononega-like virus 1 have previously been described in mosquitoes [12,15,29]. Although abundant, the novel Gysinge virus did not cluster with any mosquito sequences (Figure 5), so its true host is uncertain.

The Qinviruses are a newly described and highly divergent group of RNA viruses [10]. We identified four novel Qin-like viruses: Nackenback virus, Gran virus, Vinslov virus, and Vittskovle virus. The latter three are more closely related to the Hubei qinvirus-like virus 2 previously found in a pool of different arthropod species [10]. Nackenback virus was found to share a more recent common ancestor with the Wilkie Qin-like virus previously found in *Aedes* and *Culex* mosquitoes in Australia [12]. Although Qin-like was most closely related to fungal viruses [12], it is notable that Nackenback virus was found in both *Cx. pipiens* and *Cx. torrentium* libraries and was also more abundant than host non-RNA in the *Cx. torrentium* libraries (Table 2, Table 3). Hence, this virus may be truly mosquito associated. We also detected two orthomyxoviruses, Wuhan Mosquito Virus 6 and Wuhan Mosquito Virus 4, both of which have previously been found in pools of *Culex* mosquitoes and are known to be mosquito associated [12].

### 3.6. Double-Stranded RNA Viruses

We identified eight double-stranded RNA viruses in our Swedish mosquitoes, all of which were novel: *Partitiviridae* (*n* = 2), *Reoviridae* (*n* = 1), and *Toti/Chrysoviridae* (*n* = 5). For the family *Reoviridae*, Valmbacken virus clustered with *Aedes camptorhynchus* reo-like virus, previously discovered in mosquitoes [12]. Valmbacken virus was also abundant and found in all libraries and is therefore most likely a *Culex* associated reovirus (Table 2, Table 3). 

In comparison, four of the five novel totiviruses clustered with other mosquito-associated totiviruses (Figure 6), but only two (Lindangsbacken virus and Eskilstorp virus) were also abundant. The fifth totivirus, Ahus virus, was highly divergent, had low abundance, and clustered distantly with potentially protist originating viruses (Figure 6). Thus, the host association of Ahus virus remains uncertain.

The two novel partiti-like viruses, Vivastbo virus and Sonnbo virus, did not cluster with any viruses sequenced from mosquitoes, but rather grouped with viruses originating from various arthropod hosts (Figure 6). However, the relatively high abundance levels of the Vivastbo virus suggest that it may be associated with mosquitoes (Table 2, Table 3). 

Finally, all sequencing libraries generated here were negative for *Wolbachia* as assessed by mapping against the COX1 and Wolbachia surface protein (WSP) genes of *Wolbachia pipientis.* Although it is not possible to completely exclude the presence of other *Wolbachia* variants, our results suggest that differential presence/absence of *Wolbachia* has not affected the observed patterns of viral diversity and abundance.

## 4. Discussion

Through total RNA-sequencing of 270 *Culex* mosquitoes collected in Sweden we identified 40 viruses, including 28 that are novel. A virome comparison between the two vector species *Cx. pipiens* and *Cx. torrentium* revealed that although these mosquitoes are from the same genus and have overlapping geographical distribution, the virome family and species composition and abundance differed to some extent between the two species, and also by geographic location, at this scale of sampling (Figure 1, Figure 2, Table 2). 

Viewed at the family/order level, the relative virome abundance of *Cx. pipiens* was dominated by luteo-, orthomyxo-, and the Nam Dinh nidovirus. In comparison, *Cx. torrentium* was dominated by the Nam Dinh nidovirus in addition to the picorna-, mononega-, and reo-viruses. It should be noted, however, that family-wide comparisons could be skewed by the presence of single highly abundant viruses, as was the case here (particularly Nam Dinh virus that represented 30% of all reads in library 2), such that analyses of relative abundance and diversity are better conducted at the species level. Viewed at the level of species per region, we identified several viruses that were seemingly unique to their respective sampling location (Figure 3). This suggests that local acquisition, as well as local ecosystem and habitat composition, may be important in shaping virome compositions, although this will need to be confirmed with a larger sample size of mosquitoes. Although we did not find any evidence that sampling year impacted the results, as the majority of viruses were found in libraries covering different sampling years (Table 2, Table S2) it is likely that detailed longitudinal sampling would provide more information on possible seasonal changes in virome composition. 

Direct comparisons between published virome studies are complicated by such factors as differences in sequencing technologies, bioinformatic analyses, criteria for species demarcation, and study focus. Despite these important caveats, it is noteworthy that the number of viruses found in the relatively small sample here is of a similar magnitude and diversity to those found at lower latitudes [12,15]. Hence, the virome composition appears not to follow the same trend as mosquito biodiversity, with fewer species in temperate regions [17,18]. Specifically, 24 different viruses were found in *Cx. torrentium*, of which six were unique to that species, and 34 viruses were found in *Cx. pipiens*, of which 16 were unique. Hence, 18 viruses were shared between both mosquito species, 16 of which we tentatively consider to be mosquito associated based on their abundance and phylogenetic position (Figure 3, Figure 4, Figure 5 and Figure 6, Table 3). 

**Figure 7:**
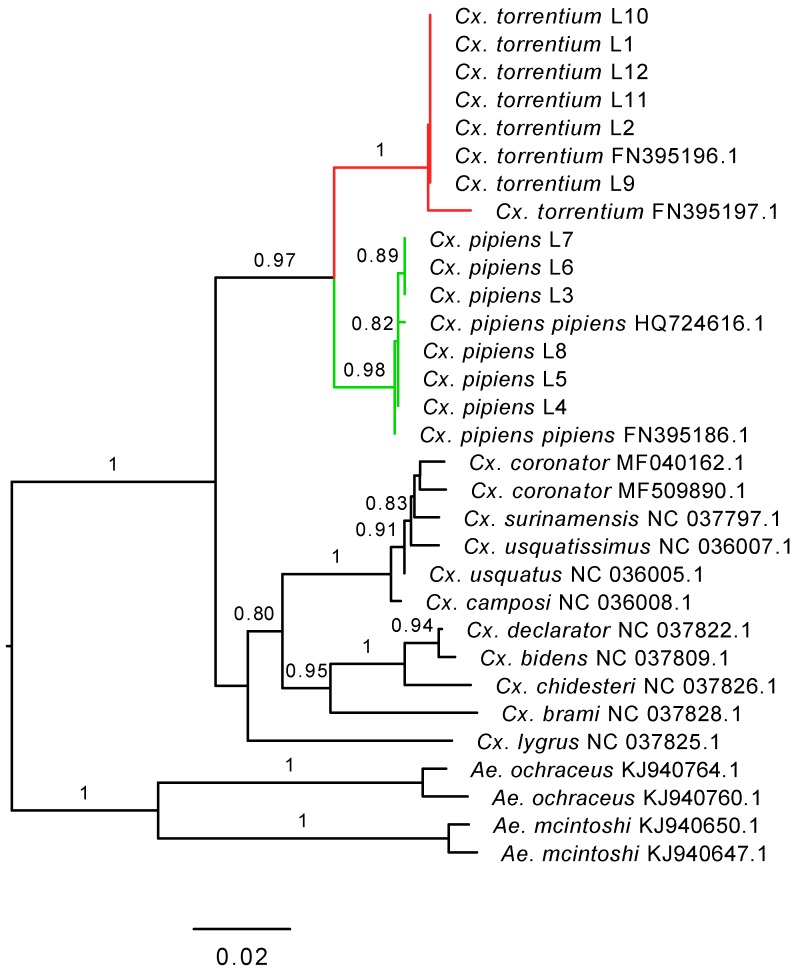
Phylogenetic relationships, based on partial COX1-gene, of *Cx. pipiens* and *Cx. torrentium* for all libraries (L1–L12) together with representative publicly available reference sequences (with their associated GenBank accession numbers). Numbers on branches indicate SH support, and only branches with SH support ≥80% are indicated. Branch lengths are scaled according to the number of nucleotide substitutions per site. The tree is midpoint rooted for clarity only.

Given their relatively close phylogenetic relationship (Figure 7), and the fact that both mosquito species inhabit the same region, share larval habitat [4], and blood-meal hosts [30], the difference in virome composition between *Cx. pipiens* and *Cx. torrentium* is striking. By considering virus abundance and phylogenetic position we suggest that 26 of the viruses discovered were likely mosquito associated (Figure 4, Figure 5 and Figure 6, Table 3), although we cannot exclude either false-negative or false-positive associations. For example, the divergent Ista virus (*Picornaviridae*) was found in high abundance and in multiple libraries but did not cluster with any viruses that originated from mosquitoes, although it did group with other arthropods (Figure 4, Figure 5 and Figure 6). The fact that it did not cluster with other mosquito viruses is perhaps unsurprising as studies from temperate regions are few, and this is the first study investigating the virome of *Cx. torrentium*. It is clear that many viruses are seemingly ubiquitous in mosquitoes, covering a wide variety of climates and habitats [12,15,31], but whether Ista virus and many other viruses are truly mosquito associated will need to be considered in additional studies. In particular, virus isolation and cell-culture/lineage experiments will be central to determining the host association of the Ista virus and other viruses discovered via meta-transcriptomic studies. It was also noteworthy that no insect-specific flaviviruses were discovered in this study, even though these are relatively commonplace [32] and have previously been found in mosquitoes in Northern Europe [33,34]. Relatedly, although we found no evidence that the virus sequences obtained here are from endogenous viral elements (EVEs) [35,36], we cannot definitively exclude that some of the viruses documented may in fact be derived from other organisms and/or host genomes present within or on the outside of the mosquito.

Notably, our study reveals that pathogenic viruses such as SINV can sometimes have similar abundance levels to viruses not associated with human disease (Table 2), in turn suggesting that pathogenic viruses form a natural part in the overall virome composition. The difference in the abundance of specific viruses between *Cx. torrentium* and *Cx. pipiens* is interesting. It has previously been shown that *Cx. pipiens* is commonly infected with *Wolbachia*, while this bacterium is absent from *Cx. torrentium* [9]. *Wolbachia* is well-known for its ability to block virus infection in some mosquito species, although it has mostly been studied in systems with pathogenic viruses such as dengue [37]. Although one potential explanation for the difference in virome composition is differential associations with the intracellular bacteria *Wolbachia pipientis*, we found no compelling evidence for *Wolbachia* in any of the *Culex* samples studied here. 

The species separation between *Cx. pipiens* and *Cx. torrentium* has long been ignored, largely because of the need for molecular demarcation, so that it has been assumed that most of the biology of the two species is comparable. Our study indicates that these two species have differing virome compositions and also that *Culex* mosquitoes in northern temperate regions can harbor similar viral diversity as mosquitoes in tropical and sub-tropical regions. Further studies should consider the host range of these viruses, their potential interactions with pathogenic viruses, and how virome composition is determined by mosquito host structure and feeding preference. Isolating the viruses discovered here will also be a key priority to enable a better understand of mosquito–virus co-evolution. Ultimately, it will be essential to identify the key evolutionary, ecological, and environmental factors that determine virome composition, as well as the impact of virome composition on the mosquito.

## Figures and Tables

**Table 1:** Overview of viral RdRp-motif library content compared to the total number of non-viral RNA reads per library.

	*Cx. torrentium*						*Cx. pipiens*					
Library	L1	L2	L9	L10	L11	L12	L3	L4	L5	L6	L7	L8
Total virus reads	311,893	22,961,076	258,016	9,604,141	4,654,540	7,452,859	45,019	279,568	565,968	57,867	227,988	186,792
Host COX1 reads	586	265	322	2427	2254	3117	126	2529	317	2850	860	417
Total reads	34,150,856	62,820,620	43,914,132	52,916,282	62,936,342	59,016,596	39,231,440	41,210,662	41,328,330	40,762,624	44,703,752	46,526,884
Virus %	0.9133	36.5502	0.5875	18.1497	7.3956	12.6284	0.1148	0.6784	1.3694	0.1420	0.5100	0.4015
Host COX1 %	0.0017	0.0004	0.0007	0.0046	0.0036	0.0053	0.0003	0.0061	0.0008	0.0070	0.0019	0.0009
Other %	99.0850	63.4494	99.4117	81.8457	92.6008	87.3663	99.8849	99.3155	98.6298	99.8510	99.4881	99.5976

**Table 2:** Individual abundance of each virus, measured as reads per million, as well as *Wolbachia* bacteria, in comparison to the abundance of the host COX1 gene.

				*Cx. torrentium*						*Cx. pipiens*					
				SINV+		SINV Unscreened				SINV+	SINV Unscreened				
			Location	Dalälven	Dalälven	Dalälven	Dalälven	Dalälven	Dalälven	Dalälven	Dalälven	Dalälven	Kristianstad	Kristianstad	Dalälven
Virus	Threshold		# Mosq	10	10	50	50	15	15	10	15	15	15	15	50
	*	**	Length	L1	L2	L9	L10	L11	L12	L3	L4	L5	L6	L7	L8
Sindbis virus	0672	1	11,688	671,784	417,443	0387	0151	0540	0474	2523	0995	0339	2012	0380	0107
Nam Dinh virus	303,146	1	20,240	73,673	303,145,830	85,644	120,821,017	28,690,339	95,244	137,084	83,376	10,006,671	116,626	87,174	106,154
Biggie virus	35,064	1	9207	16,720	15,525,826	15,439	34,543,772	23,405	35,063,781	46,391	18,709	20,978	18,301	21,363	28,930
Negev virus	9715	1	9493	1171	9,715,234	1070	2192	1748	1915	2651	1432	1670	2723	1946	582,029
Rinkaby virus	0706	1	14,498	0000	0159	0068	0151	0127	0034	0280	0000	0387	0196	706,361	0172
Kerstinbo virus	1825	1	11,280	038	0207	0250	10,734	117,913	0136	156,456	1,825,401	0266	0098	146,475	20,633
Forneby virus	0138	1	8255	0000	0064	0000	3288	0000	0000	0051	138,241	0000	0049	0000	6362
Osterfarnebo virus	0087	1	5357	0000	0000	0046	1077	0000	0000	0000	87,016	0000	0000	0000	4900
Hallsjon virus	0014	1	2847	0000	0000	0000	0491	13,712	0000	0000	0340	0048	0442	0000	0000
Tarnsjo virus (variant 1)	0063	1	7890	49,047	0000	11,955	27,629	63,064	2864	0051	0000	0024	14,253	0045	0086
Tarnsjo virus (variant 2)	0076	1	7838	7701	0000	2095	3553	10,471	0491	0000	0000	0048	76,075	0000	0021
Culex associated luteo like virus	1101	1	2790	0322	0127	0182	0113	0127	0322	0510	0170	0387	0368	1,101,496	109,421
Berrek virus	0014	1	2807	0000	0000	0046	0000	0000	0034	0000	0000	0000	0000	0000	13,906
Fagle virus	0002	1	1453	0000	1544	0000	0000	0000	0000	0000	0000	0000	0000	0000	0000
Marma virus	2602	1	3151	0703	0493	0934	1077	0604	0729	1963	0849	0919	1251	1,737,371	2,601,593
Merida virus	12,423	1	11,785	2,327,789	2,408,604	2,230,876	7,176,373	7420	12,423,082	16,084	9803	2,037,222	6526	6219	301,911
Culex mononega like virus 2	1216	1	13,316	467,748	157,082	285,831	1,216,374	553,242	311,692	1096	467,088	87,857	13,296	0761	23,513
Gysinge virus	0260	1	9532	0088	0032	0091	0170	7245	0102	115,749	259,544	0073	0147	68,093	0086
Culex mosquito virus 4	0453	1	11,954	0059	452,511	0068	1928	0079	1881	0102	0000	0097	0270	0045	0129
Culex mononega like virus 1	0083	1	6604	0000	0000	0046	0038	0000	0000	0000	0000	0000	0000	0000	83,113
Valmbacken virus	52,265	1	4315	19,092	32,360,489	139,363	1,158,301	29,172	52,264,756	66,401	24,678	21,462	19,331	20,983	30,993
Jotan virus	20,309	1	9112	16,808	18,433	15,712	7,758,198	41,738,015	20,308,796	50,878	91,457	912,788	18,718	18,119	22,224
Ista virus	5605	1	9551	5,605,247	576,435	2,529,573	7,361,250	2,402,094	3,661,343	8131	8129	4065	3950	4094	116,771
Wuhan Mosquito Virus 4	1900	1	2445	1318	229,558	520,561	642,373	332,654	1,679,358	677,569	1,900,406	1742	325,740	930,772	70,003
Wuhan Mosquito Virus 6	0636	1	2440	0117	0127	0774	0189	0127	0102	0102	0097	636,319	510,664	2349	1891
Vivastbo virus	1338	1	2157	0000	0096	0091	34,791	3972	0169	7341	1,338,440	0266	0393	119,475	26,974
Sonnbo virus	0088	1	1737	0000	0032	0000	0038	0064	0034	0000	87,720	0048	0000	0000	15,561
Rasbo virus	0017	1	5974	0000	0000	4964	7257	4798	17,368	0051	0049	0000	0000	0000	0043
Kristianstad virus	0022	1	5406	0000	0000	0000	0038	0000	0000	0000	0000	0000	22,104	0000	0000
Asum virus	0362	1	7184	0000	0032	0410	0397	0016	0051	0051	0218	0097	361,949	0045	0043
Salari virus	0014	1	6630	0000	0000	0000	0000	0000	0000	0051	0000	0000	0000	0045	13,906
Anjon virus	0407	1	6495	3485	406,952	140,775	725,051	6864	537,290	0918	0582	1089	0589	0224	0645
Gran virus	0012	1	5622	0000	0032	9473	5858	3559	11,912	0000	0000	0048	0000	0000	0021
Nackenback virus	0559	1	6128	0059	0064	0091	0113	8374	0102	0102	559,443	0048	0123	65,498	0107
Vinslov virus	0047	1	5590	0000	0000	0046	0000	0000	0000	0000	0340	8082	47,445	0000	0000
Vittskovle virus	0019	1	5671	0000	0684	0046	0000	0000	0000	0000	0000	0000	19,380	0000	0000
Ahus virus	0027	1	7732	0000	0032	0000	0038	0000	0000	0000	0243	4404	26,691	0000	0000
Osta virus	0019	1	5398	0000	0032	0000	0000	0000	0000	0102	19,364	0000	0049	10,111	0043
Lindangsbacken virus	0105	1	6171	0000	104,711	0046	0000	0000	0000	0000	0000	0000	0000	0045	0000
Salja virus	0002	1	1286	0000	0000	0000	0000	0000	0000	0000	1626	0000	0000	1611	0000
Eskilstorp virus	0210	1	2933	0000	0000	0000	0076	0032	0068	0076	0000	0000	0049	210,363	0129
Wolbachia COX1	0001	1	1573	000	0.99	000	000	000	000	000	0.05	0.05	000	000	000
Wolbachia WSP	0000	1	614	000	0,00	000	000	0.03	0.20	0.10	0.34	0.22	000	000	0.09
Host COX1	0067	1	1506	17.16	4.22	7.33	4.86	35.81	52.82	14.94	6.43	7.79	59.54	50.42	66.99
Number of virus species	–	–	–	6	13	9	17	14	12	10	13	8	10	12	17

* = 0.1% of the most abundant library; ** = Less than 1 per million reads; Grey = not present; Green = present, but not abundant; Orange = present and >0.1% of total reads; Red = present and more abundant than host; SINV+ = Mosquito pool is positive for Sindbis virus (SINV); SINV unscreened = mosquito pool has not been screened for SINV.

**Table 3:** Indication of host associations for the viruses discovered here. Likely host association was assessed using (i) the abundance of viral contig per total amount of reads in a library, (ii) virus abundance in relation to the host COX1 gene, (iii) presence across libraries, and (iv) phylogenetic clustering with other mosquito-derived viruses. If a particular virus met two or more of the four criteria, it was considered as a mosquito-associated virus.

Virus	Virus Family	*Cx. torrentium*	*Cx. pipiens*	Abundant?	More Abundant than Host RNA?	Present in >2 Libraries?	Clusters with Mosquito Viruses?	Mosquito Associated?
Sindbis virus	Alpha	P	P	Yes	Yes	No	Yes	Yes
Nam Dinh virus	Nido	P	P	Yes	Yes	Yes	Yes	Yes
Biggie virus	Virga–Negev	P	P	Yes	Yes	Yes	Yes	Yes
Negev virus	Virga–Negev	P	P	Yes	Yes	No	Yes	Yes
Rinkaby virus	Virga–Negev	NP	P	Yes	Yes	No	No	Yes
Kerstinbo virus	Endorna	P	P	Yes	Yes	No	No	Yes
Forneby virus	Endorna	P	P	No	Yes	No	No	No
Osterfarnebo virus	Endorna	P	P	No	Yes	No	No	No
Hallsjon virus	Endorna	P	NP	No	No	No	No	No
Tarnsjo virus (variant 1)	Tymo	P	P	No	Yes	No	Yes	Yes
Tarnsjo virus (variant 2)	Tymo	P	P	No	Yes	No	Yes	Yes
Culex associated luteo like virus	Luteo	NP	P	Yes	Yes	No	Yes	Yes
Berrek virus	Luteo	NP	P	No	No	No	Yes	No
Fagle virus	Luteo	P	NP	No	No	No	No	No
Marma virus	Luteo	NP	P	Yes	Yes	No	Yes	Yes
Merida virus	Mononega	P	P	Yes	Yes	Yes	Yes	Yes
Culex mononega like virus 2	Mononega	P	P	Yes	Yes	Yes	Yes	Yes
Gysinge virus	Mononega	P	P	Yes	Yes	Yes	No	Yes
Culex mosquito virus 4	Mononega	P	NP	Yes	Yes	No	Yes	Yes
Culex mononega like virus 1	Mononega	NP	P	No	Yes	No	Yes	Yes
Valmbacken virus	Reo	P	P	Yes	Yes	Yes	Yes	Yes
Jotan virus	Picorna	P	P	Yes	Yes	Yes	Yes	Yes
Ista virus	Picorna	P	P	Yes	Yes	Yes	No	Yes
Wuhan Mosquito Virus 6	Orthomyxo	P	P	Yes	Yes	Yes	Yes	Yes
Wuhan Mosquito Virus 4	Orthomyxo	NP	P	Yes	No	No	Yes	Yes
Vivastbo virus	Partiti	P	P	Yes	Yes	No	No	Yes
Sonnbo virus	Partiti	NP	P	No	Yes	No	No	No
Rasbo virus	Bunya	P	NP	No	No	No	No	No
Kristianstad virus	Bunya	NP	P	No	No	No	Yes	No
Asum virus	Bunya	NP	P	Yes	No	No	No	No
Salari virus	Bunya	NP	P	No	No	No	Yes	No
Anjon virus	Phasma	P	P	Yes	Yes	Yes	Yes	Yes
Gran virus	Qin	P	NP	No	No	No	No	No
Nackenback virus	Qin	P	P	Yes	Yes	Yes	No	Yes
Vinslov virus	Qin	NP	P	No	No	No	No	No
Vittskovle virus	Qin	NP	P	No	No	No	No	No
Ahus virus	Toti	NP	P	No	No	No	No	No
Osta virus	Toti	NP	P	No	No	No	Yes	No
Lindangsbacken virus	Toti	P	NP	No	Yes	No	Yes	Yes
Salja virus	Toti	NP	P	No	No	No	Yes	No
Eskilstorp virus	Chryso	NP	P	No	Yes	No	Yes	Yes

P = present; NP = not present; >0.1% = abundant.

## Supplementary Materials

The following are available online at https://www.mdpi.com/1999-4915/11/11/1033/s1, Table S1: Information on collection site, year of collection and pool size for all *Cx. pipiens* and *Cx. torrentium* libraries; Table S2: Number of reads mapped to each virus, *Wolbachia* and host genes per library per mosquito species.

## References

[1] Mullen G.R., Durden L. (2009). Medical and Veterinary Entomology.

[2] Gould E., Pettersson J., Higgs S., Charrel R., de Lamballerie X. (2017). Emerging arboviruses: Why today?. One Health.

[3] Weaver S.C., Lecuit M. (2015). Chikungunya virus and the global spread of a mosquito-borne disease. N. Engl. J. Med..

[4] Hesson J.C., Rettich F., Merdić E., Vignjević G., Ostman O., Schäfer M., Schaffner F., Foussadier R., Besnard G., Medlock J. (2014). The arbovirus vector *Culex torrentium* is more prevalent than Culex pipiens in northern and central Europe. Med. Vet. Entomol..

[5] Kurkela S., Helve T., Vaheri A., Vapalahti O. (2008). Arthritis and arthralgia three years after Sindbis virus infection: Clinical follow-up of a cohort of 49 patients. Scand. J. Infect. Dis..

[6] Hesson J.C., Verner-Carlsson J., Larsson A., Ahmed R., Lundkvist Å., Lundström J.O. (2015). Culex torrentium Mosquito Role as Major Enzootic Vector Defined by Rate of Sindbis Virus Infection, Sweden, 2009. Emerg. Infect. Dis..

[7] Hesson J.C., Lundström J.O., Tok A., Östman Ö., Lundkvist Å. (2016). Temporal variation in Sindbis virus antibody prevalence in bird hosts in an endemic area in Sweden. PLoS ONE.

[8] Komar N., Langevin S., Hinten S., Nemeth N., Edwards E., Hettler D., Davis B., Bowen R., Bunning M. (2003). Experimental infection of North American birds with the New York 1999 strain of West Nile virus. Emerg. Infect. Dis..

[9] Leggewie M., Krumkamp R., Badusche M., Heitmann A., Jansen S., Schmidt-Chanasit J., Tannich E., Becker S.C. (2018). Culex torrentium mosquitoes from Germany are negative for Wolbachia. Med. Vet. Entomol..

[10] Shi M., Lin X.-D., Tian J.-H., Chen L.-J., Chen X., Li C.-X., Qin X.-C., Li J., Cao J.-P., Eden J.-S. (2016). Redefining the invertebrate RNA virosphere. Nature.

[11] Shi M., Lin X.-D., Chen X., Tian J.-H., Chen L.-J., Li K., Wang W., Eden J.-S., Shen J.-J., Liu L. (2018). The evolutionary history of vertebrate RNA viruses. Nature.

[12] Shi M., Neville P., Nicholson J., Eden J.-S., Imrie A., Holmes E.C. (2017). High-Resolution Metatranscriptomics Reveals the Ecological Dynamics of Mosquito-Associated RNA Viruses in Western Australia. J. Virol..

[13] Atoni E., Wang Y., Karungu S., Waruhiu C., Zohaib A., Obanda V., Agwanda B., Mutua M., Xia H., Yuan Z. (2018). Metagenomic virome analysis of Culex mosquitoes from Kenya and China. Viruses.

[14] Li C.-X., Shi M., Tian J.-H., Lin X.-D., Kang Y.-J., Chen L.-J., Qin X.-C., Xu J., Holmes E.C., Zhang Y.-Z. (2015). Unprecedented genomic diversity of RNA viruses in arthropods reveals the ancestry of negative-sense RNA viruses. eLife.

[15] Sadeghi M., Altan E., Deng X., Barker C.M., Fang Y., Coffey L.L., Delwart E. (2018). Virome of >12 thousand Culex mosquitoes from throughout California. Virology.

[16] Zhang W., Li F., Liu A., Lin X., Fu S., Song J., Liu G., Shao N., Tao Z., Wang Q. (2018). Identification and genetic analysis of Kadipiro virus isolated in Shandong province, China. Virol. J..

[17] Brown J.H. (2014). Why are there so many species in the tropics?. J. Biogeogr..

[18] Foley D.H., Rueda L.M., Wilkerson R.C. (2007). Insight into global mosquito biogeography from country species records. J. Med. Entomol..

[19] Becker N., Petrić D., Boase C., Lane J., Zgomba M., Dahl C., Kaiser A. (2003). Mosquitoes and Their Control.

[20] Hesson J.C., Lundström J.O., Halvarsson P., Erixon P., Collado A. (2010). A sensitive and reliable restriction enzyme assay to distinguish between the mosquitoes *Culex torrentium* and *Culex pipiens*. Med. Vet. Entomol..

[21] Bolger A.M., Lohse M., Usadel B. (2014). Trimmomatic: A flexible trimmer for Illumina sequence data. Bioinformatics.

[22] Haas B.J., Papanicolaou A., Yassour M., Grabherr M., Blood P.D., Bowden J., Couger M.B., Eccles D., Li B., Lieber M. (2013). De novo transcript sequence reconstruction from RNA-seq using the Trinity platform for reference generation and analysis. Nat. Prot..

[23] Buchfink B., Xie C., Huson D.H. (2015). Fast and sensitive protein alignment using DIAMOND. Nat. Methods.

[24] Langmead B., Salzberg S.L. (2012). Fast gapped-read alignment with Bowtie 2. Nat. Methods.

[25] Pettersson J.H.-O., Shi M., Bohlin J., Eldholm V., Brynildsrud O.B., Paulsen K.M., Andreassen Å., Holmes E.C. (2017). Characterizing the virome of Ixodes ricinus ticks from northern Europe. Sci. Rep..

[26] Katoh K., Standley D.M. (2013). MAFFT multiple sequence alignment software version 7: Improvements in performance and usability. Mol. Biol. Evol..

[27] Guindon S., Dufayard J.F., Lefort V., Anisimova M., Hordijk W., Gascuel O. (2010). New algorithms and methods to estimate maximum-likelihood phylogenies: Assessing the performance of PhyML 3.0. Syst. Biol..

[28] Marzano S.-Y.L., Domier L.L. (2016). Novel mycoviruses discovered from metatranscriptomics survey of soybean phyllosphere phytobiomes. Virus Res..

[29] Charles J., Firth A.E., Loroño-Pino M.A., Garcia-Rejon J.E., Farfan-Ale J.A., Lipkin W.I., Blitvich B.J., Briese T. (2016). Merida virus, a putative novel rhabdovirus discovered in Culex and *Ochlerotatus* spp. mosquitoes in the Yucatan Peninsula of Mexico. J. Gen. Virol..

[30] Hesson J.C. Bloodmeal analyses of Sindbis virus vectors.

[31] Ling J., Smura T., Lundström J.O., Pettersson J.H.-O., Sironen T., Vapalahti O., Lundkvist Å., Hesson J.C. (2019). The introduction and dispersal of Sindbis virus from central Africa to Europe. J. Virol..

[32] Blitvich B.J., Firth A.E. (2015). Insect-specific flaviviruses: A systematic review of their discovery, host range, mode of transmission, superinfection exclusion potential and genomic organization. Viruses.

[33] Huhtamo E., Putkuri N., Kurkela S., Manni T., Vaheri A., Vapalahti O., Uzcategui N.Y. (2009). Characterization of a novel flavivirus from mosquitoes in northern Europe that is related to mosquito-borne flaviviruses of the tropics. J. Virol..

[34] Huhtamo E., Moureau G., Cook S., Julkunen O., Putkuri N., Kurkela S., Uzcátegui N.Y., Harbach R.E., Gould E.A., Vapalahti O. (2012). Novel insect-specific flavivirus isolated from northern Europe. Virology.

[35] Olson K.E., Bonizzoni M. (2017). Nonretroviral integrated RNA viruses in arthropod vectors: An occasional event or something more?. Curr. Opin. Insect Sci..

[36] Cui J., Holmes E.C. (2012). Endogenous RNA viruses of plants in insect genomes. Virology.

[37] Johnson K.N. (2015). The Impact of Wolbachia on Virus Infection in Mosquitoes. Viruses.

