# Supplementary material for: Meta-Transcriptomic Comparison of the RNA Viromes of the Mosquito Vectors Culex pipiens and Culex torrentium in Northern Europe
Source: Viruses. 2019 Nov 6;11(11):1033. doi: 10.3390/v11111033 (PMC6893722; doi:10.3390/v11111033)
Supplement: Supplementary file 1 [file viruses-11-01033-s001.pdf]

Supplementary Table S1. Information on collection site, year of collection and pool size for all *Cx. pipiens* and *Cx. torrentium* libraries.

Pool/Library	Species	Number of mosquitoes	Region	Year
L1	Cx. torrentium	10	Dalälven	2009
L2	Cx. torrentium	10	Dalälven	2009
L3	Cx. pipiens	10	Dalälven	2009
L4	Cx. pipiens	15	Dalälven	2011
L5	Cx. pipiens	15	Dalälven	2006
L6	Cx. pipiens	15	Kristianstad	2006
L7	Cx. pipiens	15	Kristianstad	2007
L8	Cx. pipiens	50	Dalälven	2009
L9	Cx. torrentium	50	Dalälven	2009
L10	Cx. torrentium	50	Dalälven	2009
L11	Cx. torrentium	15	Dalälven	2011
L12	Cx. torrentium	15	Dalälven	2006

Supplementary Table S2. Number of reads mapped to each virus, *Wolbachia* and host genes per library per mosquito species.

		Culex torrentium								Culex pipiens							
		SINV+		SINV unscreened						SINV+	SINV unscreened						
		Dalälven	Dalälven	Dalälven	Dalälven	Dalälven	Dalälven			Dalälven	Dalälven	Dalälven	Kristianstad	Kristianstad	Dalälven		
**Virus**	**Lenght**	**L1**	**L2**	**L9**	**L10**	**L11**	**L12**	**Total reads**	**Virus fam**	**L3**	**L4**	**L5**	**L6**	**L7**	**L8**	**reads**	**Virus fam**
Sindbis virus	11688	22942	26224	17	8	34	28	49253	Alpha	99	41	14	82	17	5	258	Alpha
Nam Dinh virus	20240	2516	19043809	3761	6393399	1805665	5621	27254771	Nido	5378	3436	413559	4754	3897	4939	435963	Nido
Biggie virus	9207	571	975342	678	1827928	1473	2069345	4875337	Virga–Negev	1820	771	867	746	955	1346	6505	Virga–Negev
Negev virus	9493	40	610317	47	116	110	113	610743	Virga–Negev	104	59	69	111	87	27080	27510	Virga–Negev
Rinkaby virus	14498	0	10	3	8	8	2	31	Virga–Negev	11	0	16	8	31577	8	31620	Virga–Negev
Kerstinbo virus	11280	13	13	11	568	7421	8	8034	Endorna	6138	75226	11	4	6548	960	88887	Endorna
Forneby virus	8255	0	4	0	174	0	0	178	Endorna	2	5697	0	2	0	296	5997	Endorna
Osterfarnebo virus	5357	0	0	2	57	0	0	59	Endorna	0	3586	0	0	0	228	3814	Endorna
Hallsjon virus	2847	0	0	0	26	863	0	889	Endorna	0	14	2	18	0	0	34	Endorna
Tarnsjo virus (variant 1)	7890	1675	0	525	1462	3969	169	7800	Tymo	2	0	1	581	2	4	590	Tymo
Tarnsjo virus (variant 2)	7838	263	0	92	188	659	29	1231	Tymo	0	0	2	3101	0	1	3104	Tymo
Culex associated luteo like virus	2790	11	8	8	6	8	19	60	Luteo	20	7	16	15	49241	5091	54390	Luteo
Berrek virus	2807	0	0	2	0	0	2	4	Luteo	0	0	0	0	0	647	647	Luteo
Fagle virus	1453	0	97	0	0	0	0	97	Luteo	0	0	0	0	0	0	0	Luteo
Marma virus	3151	24	31	41	57	38	43	234	Luteo	77	35	38	51	77667	121044	198912	Luteo
Merida virus	11785	79496	151310	97967	379747	467	733168	1442155	Mononega	631	404	84195	266	278	14047	99821	Mononega
Culex mononega like virus 2	13316	15974	9868	12552	64366	34819	18395	155974	Mononega	43	19249	3631	542	34	1094	24593	Mononega
Gysinge virus	9532	3	2	4	9	456	6	480	Mononega	4541	10696	3	6	3044	4	18294	Mononega
Culex mosquito virus 4	11954	2	28427	3	102	5	111	28650	Mononega	4	0	4	11	2	6	27	Mononega
Culex mononega like virus 1	6604	0	0	2	2	0	0	4	Mononega	0	0	0	0	0	3867	3867	Mononega
Valmbacken virus	4315	652	2032906	6120	61293	1836	3084488	5187295	Reo	2605	1017	887	788	938	1442	7677	Reo
Jotan virus	9112	574	1158	690	410535	2626838	1198556	4238351	Picorna	1996	3769	37724	763	810	1034	46096	Picorna
Ista virus	9551	191424	36212	111084	389530	151179	216080	1095509	Picorna	319	335	168	161	183	5433	6599	Picorna
Wuhan Mosquito Virus 4	2445	45	14421	22860	33992	20936	99110	191364	Orthomyxo	26582	78317	72	13278	41609	3257	163115	Orthomyxo
Wuhan Mosquito Virus 6	2440	4	8	34	10	8	6	70	Orthomyxo	4	4	26298	20816	105	88	47315	Orthomyxo
Vivastbo virus	2157	0	6	4	1841	250	10	2111	Partiti	288	55158	11	16	5341	1255	62069	Partiti
Sonnbo virus	1737	0	2	0	2	4	2	10	Partiti	0	3615	2	0	0	724	4341	Partiti
Rasbo virus	5974	0	0	218	384	302	1025	1929	Bunya	2	2	0	0	0	2	6	Bunya
Kristianstad virus	5406	0	0	0	2	0	0	2	Bunya	0	0	0	901	0	0	901	Bunya
Asum virus	7184	0	2	18	21	1	3	45	Bunya	2	9	4	14754	2	2	14773	Bunya
Salari virus	6630	0	0	0	0	0	0	0	Bunya	2	0	0	0	2	647	651	Bunya
Anjon virus	6495	119	25565	6182	38367	432	31709	102374	Phasma	36	24	45	24	10	30	169	Phasma
Gran virus	5622	0	2	416	310	224	703	1655	Qin	0	0	2	0	0	1	3	Qin
Nackenback virus	6128	2	4	4	6	527	6	549	Qin	4	23055	2	5	2928	5	25999	Qin
Vinslov virus	5590	0	0	2	0	0	0	2	Qin	0	14	334	1934	0	0	2282	Qin
Vittskovle virus	5671	0	43	2	0	0	0	45	Qin	0	0	0	790	0	0	790	Qin
Ahus virus	7732	0	2	0	2	0	0	4	Toti	0	10	182	1088	0	0	1280	Toti
Osta virus	5398	0	2	0	0	0	0	2	Toti	4	798	0	2	452	2	1258	Toti
Lindangsbacken virus	6171	0	6578	2	0	0	0	6580	Toti	0	0	0	0	2	0	2	Toti
Salja virus	1286	0	0	0	0	0	0	0	Toti	0	67	0	0	72	0	139	Toti
Eskilstorp virus	2933	0	0	0	4	2	4	10	Chryso	3	0	0	2	9404	6	9415	Chryso
Wolbachia COX1	1573	0	62	0	0	0	0	62	Wolbachia	0	2	2	0	0	0	4	Wolbachia
Wolbachia WSP	614	0	0	0	0	2	12	14	Wolbachia	4	14	9	0	0	4	31	Wolbachia
Host COX1	1506	586	265	322	2427	2254	3117	8971	COX1	126	2529	317	2850	860	417	7099	COX1

Library	L1	L2	L9	L10	L11	L12	L3	L4	L5	L6	L7	L8
Total reads	34150856	62820620	43914132	52916282	62936342	59016596	39231440	41210662	41328330	40762624	44703752	46526884
Virus reads	316350	22962373	263351	9604522	4658534	7458761	50717	285415	568159	65620	235207	194595
0.1% of total	3415	6282	4391	5292	6294	5902	3923	4121	4133	4076	4470	4653

	**L1**	**L2**	**L9**	**L10**	**L11**	**L12**		**L3**	**L4**	**L5**	**L6**	**L7**	**L8**
**Percent virus**	**0,93**	**36,55**	**0,60**	**18,15**	**7,40**	**12,64**	**Percent virus**	**0,13**	**0,69**	**1,37**	**0,16**	**0,53**	**0,42**
**Percent Cx COX1**	**0,0017**	**0,0004**	**0,0007**	**0,0046**	**0,0036**	**0,0053**	**Percent Cx COX1**	**0,0003**	**0,0061**	**0,0008**	**0,0070**	**0,0019**	**0,0009**
**Percent other**	**99,07**	**63,45**	**99,40**	**81,85**	**92,60**	**87,36**	**Percent other**	**99,87**	**99,31**	**98,63**	**99,84**	**99,47**	**99,58**
SUM	100,00	100,00	100,00	100,00	100,00	100,00	SUM	100,00	100,00	100,00	100,00	100,00	100,00

SINV+ = Mosquito pool is positive for SINV
SINV unscreened = Mosquito pool has not been screened for SINV
